# A successful first year: taking stock and looking ahead

**DOI:** 10.2349/biij.2.2.e37

**Published:** 2006-04-01

**Authors:** KH Ng, BJJ Abdullah

**Affiliations:** Department of Biomedical Imaging (Radiology), Faculty of Medicine, University of Malaya, Kuala Lumpur, Malaysia

The biij will shortly be completing its first year of publication. This calls for a celebration for very many reasons. There have been mistakes made, lessons learned, triumphs and achievements…all in these 12 months. With this issue, we decided it was time to pause a little and take stock of our progress during the past year.

We firmly believe that knowledge should not be the monopoly of a few select individuals especially when it has the potential to enhance the quality of life for mankind. We also believe that this can be achieved through participation in the open access initiative [1-3].

One of the founding principles of the biij is to ensure that biij offers easy access to research for academics, scientists and anybody else who seek it [1]. We are fully committed to the open access model of scholarly communication, which brings benefits to authors, readers, teachers, scholars, and researchers alike. It serves as a catalyst to the research world by ensuring that new developments and discoveries reach the farthest shores.

To achieve this we have to acknowledge the support from researchers, reviewers, authors and readers who believed in our vision for the journal and helped realise it through their regular, invaluable contributions.

We also acknowledge our sponsors who have been generous and enormously supportive. It takes a huge load off our shoulders when we know that each issue of the biij will see light of day thanks to their commitment.

They say, the first year is always the most difficult to get through. The journal has been able to efficiently maintain the entire editorial lifecycle within a reasonable timeframe (data as of 29 June 2006): average time from submission to first review was 20.7 days, time lag between submission and acceptance (turn around time) was 109.9 days and a manuscript was published within a mean period of 35.2 days following acceptance. Each manuscript is processed for dual formats: a multimedia enabled, display independent web format, and a more traditional typeset PDF format. Abstracts are extracted and automatically indexed in supported bibliographic indexing engines. The number of submissions has reached 82 and our acceptance rate is currently 80.8%.

The readership of biij comes from more than 60 countries, from Canada to Cyprus, from Singapore to Slovenia, and from United States to Uruguay; making it a truly international journal. Another achievement is that we have been indexed by the following bibliographic agencies: Chemical Abstract, INSPEC, Index Copernicus, Directory of Open Access Journals and CrossRef. We aim to get indexed by Medline in the near future.

**Figure 1:**
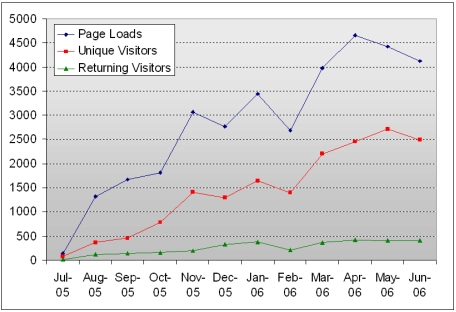
Chart shows the monthly statistics of page loads, unique visitors and returning visitors for biij, from July 2005 to June 2006.

With every passing month, the website's popularity has increased and its virtual readership has grown steadily (Figure 1). Between July 2005 and June 2006, there has been an average of 2,724.8 page loads, made by 1,345.6 unique visitors and 248.3 returning visitors each month (data retrieved from Google Analytics as of 29 June 2006).

Every article is assigned a unique Document Object Identifier (DOI) and carries the citation information which is a great boon to contributors to determine how frequently their article has been cited. The journal also provides a list of its "most-viewed articles" with the highest having reached 2,172 page loads (as of 29 June 2006).

Digital recordings of lectures and conferences have been a useful tool in learning [4], and biij has recorded many such lectures and presentations of conferences and workshops around the region. These digital video files, which are available on the biij site (http://www.biij.org/biomedical-imaging-intervention-journal-resources.asp), have proven to be a valuable educational resource.

We are planning a few special focus issues, including PET/CT and molecular imaging, image-guided surgery and therapy, radiation dose optimisation in biomedical imaging and intervention, leadership and management. To ensure a publication of the highest quality, well known researchers and practitioners have been invited to contribute articles.

Finally, we would like to especially thank our readers whose support is what keeps the journal going for each issue. Your readership, feedback, and encouragement help sustain our work and in return we assure you that the biij will strive to maintain the highest publication quality.

We can only march forward now.
